# Heat stress in the Caribbean: Climatology, drivers, and trends of human biometeorology indices

**DOI:** 10.1002/joc.7774

**Published:** 2022-07-15

**Authors:** Claudia Di Napoli, Theodore Allen, Pablo A. Méndez‐Lázaro, Florian Pappenberger

**Affiliations:** ^1^ School of Agriculture, Policy and Development University of Reading Reading UK; ^2^ Department of Geography and Environmental Science University of Reading Reading UK; ^3^ European Centre for Medium Range Weather Forecasts Reading UK; ^4^ Caribbean Institute for Meteorology and Hydrology St James Barbados; ^5^ Environmental Health Department, Graduate School of Public Health University of Puerto Rico San Juan Puerto Rico

**Keywords:** bioclimatology, Caribbean, climate adaptation, climate change, heat hazards, human health, preparedness, resilience

## Abstract

Forty years (1980–2019) of reanalysis data were used to investigate climatology and trends of heat stress in the Caribbean region. Represented via the Universal Thermal Climate Index (UTCI), a multivariate thermophysiological‐relevant parameter, the highest heat stress is found to be most frequent and geographically widespread during the rainy season (August, September, and October). UTCI trends indicate an increase of more than 0.2°C·decade^−1^, with southern Florida and the Lesser Antilles witnessing the greatest upward rates (0.45°C·decade^−1^). Correlations with climate variables known to induce heat stress reveal that the increase in heat stress is driven by increases in air temperature and radiation, and decreases in wind speed. Conditions of heat danger, as depicted by the heat index (HI), have intensified since 1980 (+1.2°C) and are found to occur simultaneously to conditions of heat stress suggesting a synergy between heat illnesses and physiological responses to heat. This work also includes the analysis of the record‐breaking 2020 heat season during which the UTCI and HI achieved above average values, indicating that local populations most likely experienced heat stress and danger higher than the ones they are used to. These findings confirm the gradual intensification of heat stress in the Caribbean and aim to provide a guidance for heat‐related policies in the region.

## Graphical abstract

Heat stress, as represented via a multivariate thermophysiological‐relevant parameter called the Universal Thermal Climate Index (UTCI), is gradually intensifying in the Caribbean region. Forty years (1980–2019) of reanalysis data reveal an increase on heat stress greater than 0.2°C·decade^−1^, especially during the rainy season (August, September, and October). A correlation analysis suggests such an increase is driven by increases in air temperature and radiation, and decreases in wind speed. These findings call for urgent heat‐related policies in the Caribbean.

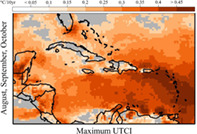

## 1 INTRODUCTION

In the recent decades heat stress has become an emerging threat in the Caribbean region with coral reefs, livestock, labour productivity, and human health being the most affected sectors (Kjellstrom *et al*., 2018; Lallo *et al*., 2018; Méndez‐Lázaro *et al*., 2018b; Muniz‐Castillo *et al*., 2019).

With regards to human health, heat stress was first identified as a major health issue for the region in 2003 (Aron *et al*., 2003). Heat stress may lead to illnesses such as sunstroke, sunburn, heat exhaustion, and dehydration (Ebi *et al*., 2006). In a survey study conducted in 2020, health professionals from 12 countries, including Jamaica, reported on heat‐related illnesses already adversely affecting local populations, and expect these illnesses to become more frequent or severe over the next 10 years (Kotcher *et al*., 2021). A 2015 focus group of Caribbean health‐care providers perceived “hotter than usual” temperatures as contributing to more hospitalizations for dehydration and sunburn in Grenada, and an increase in respiratory‐related illnesses in Trinidad and Tobago (Macpherson and Akpinar‐Elci, 2015). Excessive heat exposure has also been linked to reduced children's learning ability at school, increased demand for cooling, and a re‐envisioning of the urban environment in eastern Caribbean states (Wilkinson *et al*., 2021).

From a climatological point of view, previous research on historical climate data confirmed an increase in the occurrence of extreme high temperatures across the region as well as in single islands, from Hispaniola to Barbados, Cuba, Puerto Rico, Trinidad, and Tobago (Singh, 1997; Naranjo‐Diaz and Centella, 1998; Peterson *et al*., 2002; Aguilar *et al*., 2005; Pérez and Jury, 2012; Beharry *et al*., 2015; Jury, 2015; Méndez‐Lázaro *et al*., 2015; Jones *et al*., 2016b; Dookie *et al*., 2018; Mohan *et al*., 2020). In 2020 the Caribbean region witnessed a record‐breaking heat season. Air temperatures set new national records in Cuba in April, and in Dominica, Grenada, and Puerto Rico in September (WMO, 2021). Being one of the three warmest years for the Caribbean region compared with the average temperature for the 1981–2010 period, 2020 placed itself in the upward trend in observed air temperatures since the mid‐1990s (Angeles‐Malaspina *et al*., 2018; WMO, 2021). As for climate projections, these foresee a further increase in the duration, intensity, and frequency of extreme heat events in the Caribbean region regardless of the future scenario, with positive trends predicted for warm days and nights (Biasutti *et al*., 2011; Campbell *et al*., 2011; Hall *et al*., 2012; Taylor *et al*., 2012; Karmalkar *et al*., 2013; McLean *et al*., 2015; Jones *et al*., 2016a; 2016b; Stennett‐Brown *et al*., 2017; Angeles‐Malaspina *et al*., 2018; Taylor *et al*., 2018).

As awareness grows on the linkages between heat and human health, a need has arisen to estimate what humans feel under different climatic conditions. Biometeorological indices have recently been used for the scope. One of these indices is the heat index (HI), which combines air temperature and relative humidity to posit a human‐perceived apparent temperature from a body's heat transfer balance model (Steadman, 1979). Historical trends and climate projections indicate increasing patterns for the HI as well as intensification of HI‐defined extreme events across the Caribbean region (Ramirez‐Beltran *et al*., 2017; Angeles‐Malaspina *et al*., 2018). More sophisticated biometeorological indices have also be investigated with the aim to evidence the relevance and impact of the climate from a human thermophysiological perspective. These indices include the Universal Thermal Climate Index (UTCI), which describes the physiological heat stress the human body experiences in the attempt to maintain a thermal equilibrium with the surrounding outdoor environment (Błażejczyk *et al*., 2013). Combining an advanced body's physiological model with a state‐of‐the‐art clothing insulation model for outdoor climates (UTCI‐Fiala model), the UTCI has long been considered superior to other indices in representing thermal conditions and their variability (Blazejczyk *et al*., 2012; Fiala *et al*., 2012; Havenith *et al*., 2012). Yet, its application in the Caribbean region remains limited and mostly focused on the assessment of bioclimatic comfort in touristic settings, such as coastal resorts (Rutty and Scott, 2014; Rutty and Scott, 2015).

The UTCI needs four climate variables—2 m air temperature, relative humidity, 10 m wind speed, and radiation—to be calculated (Brode *et al*., 2012). This has been perceived as a limiting factor from local stakeholders, as most of environmental data, especially those from ground meteorological stations, are unavailable (Climate Studies Group Mona, 2020). Maintaining a consistent and continuous daily recordkeeping of ground weather observations has historically been considered challenging in the Caribbean region, leading to gaps in the recorded time series as well as in those geographical areas where meteorological stations are lacking (Peterson *et al*., 2002). This limitation can be overcome by using climate reanalysis data. Obtained by applying forecast models and data assimilation systems on past observations, a climate reanalysis provides a complete and consistent description of the Earth's climate and its evolution in recent decades in the form of gridded time‐stepped data (Dee *et al*., 2016). To the authors' knowledge, only a few studies so far have used climate reanalysis data to investigate historical heat stress in the Caribbean region and none of them makes use of the UTCI (Ramirez‐Beltran *et al*., 2017; Angeles‐Malaspina *et al*., 2018).

In view of this, the present paper aims to assess climatology and trends of human heat stress in the Caribbean region by means of climate reanalysis data. Using ERA5‐HEAT, a derived product from the ERA5 climate reanalysis dataset (Di Napoli *et al*., 2021a), pan‐Caribbean maps and temporal trends were obtained for the UTCI over a 40‐year historical period and are presented here for the first time. The paper also examines the climate variables known to contribute to human heat stress, namely 2 m air temperature, relative humidity, 10 m wind speed, and mean radiant temperature. Called *heat stress drivers* hereafter, their climatology and trends are also investigated over the same period. As the HI has previously been used as an indicator of heat stress for the region, climatology and trends of the HI are included and discussed against the UTCI. Finally, the extreme heat episode affecting the Caribbean region in 2020 is examined.

## 2 DATA AND METHODS

Reanalysis data served as the basis for (a) estimating the thermal bioclimate of the Caribbean region in terms of human heat stress and (b) describing the spatial and temporal variability of the UTCI, HI, and heat stress drivers across the same area.

### 2.1 Study area

**FIGURE 1:**
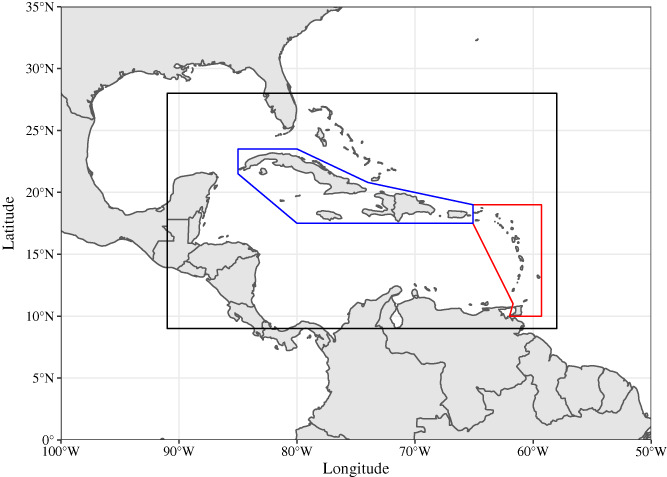
Map showing the Caribbean region considered in this study (black rectangle). Blue and red lines delimit the Greater Antilles and the Lesser Antilles study areas, respectively [Colour figure can be viewed at wileyonlinelibrary.com]

The Caribbean region encompasses large (Greater Antilles) and small (Lesser Antilles) islands as well as coastlines along Central and South America. Figure 1 shows the geographical area considered in the present study. It includes the Caribbean Sea, parts of Central America and Florida, and the northern part of South America (9°–28°N and 91°–58°W). To capture climatic variability within the Caribbean, two subregions corresponding to the Greater Antilles and the Lesser Antilles are considered. They are defined as shown in Figure 1.

### 2.2 Heat metrics

The present study focuses on the UTCI, a thermal stress metrics that has been evaluated and studied over a variety of climate regions as well as spatial and temporal scales (Coccolo *et al*., 2016). The UTCI is valid across the entire range of thermal exposure, that is, from cold to hot, and is classified into a 10‐category scale (Table 1). Each category corresponds to a well‐defined set of human physiological responses to the outdoor environment, which can be controlled and/or prevented by adopting specific behavioural and clothing styles (Havenith and Fiala, 2015). The UTCI can be calculated solving either the UTCI‐Fiala model or the UTCI operational procedure. The former may be computationally intensive and time‐consuming, so the latter is generally preferred and is here used. The operational procedure computes the offset between the UTCI and 2 m air temperature via a 6‐order polynomial equation in the climate variables of 2 m air temperature, relative humidity, 10 m wind speed, and mean radiant temperature. The procedure approximates the UTCI‐Fiala model within a root‐mean‐square error of 1.1°C (Brode *et al*., 2012).

**TABLE 1:** Category scales of the UTCI and HI, and corresponding physiological responses

Heat metrics	Range (°C)	Category	Physiological responses
Universal Thermal Climate Index (UTCI)	Above 46	Extreme heat stress	Increase in rectal temperature time gradient Steep decrease in total net heat loss Averaged sweat rate >650 g·hr^−1^, steep increase
	38–46	Very strong heat stress	Low core to skin temperature gradient Increase in rectal temperature at 30 min
	32–38	Strong heat stress	Averaged sweat rate >200 g·hr^−1^ Increase in rectal temperature at 120 min Instantaneous change in skin temperature
	26–32	Moderate heat stress	Change of slopes in sweat rate, rectal and skin (mean, face, hand) temperature Occurrence of sweating at 30 min Steep increase in skin wettedness
	9–26	No thermal stress	Averaged sweat rate >100 g·hr^−1^ Plateau in rectal temperature time gradient
	0–9	Slight cold stress	Local minimum of hand skin temperature
	−13 to 0	Moderate cold stress	Vasoconstriction Face skin temperature at 30 min < 15°C (pain)
	−13 to −27	Strong cold stress	Numbness Increase in core to skin temperature gradient
	−27 to −40	Very strong cold stress	Frostbite, numbness, shivering Steeper decrease in rectal temperature
	Below −40	Extreme cold stress	Frostbite Decrease in rectal temperature time gradient
Heat Index (HI)	Below 26.7	No caution	None
	26.7–32.2	Caution	Fatigue possible with prolonged exposure and/or physical activity
	32.2–39.4	Extreme caution	Heat stroke, heat cramps, or heat exhaustion possible with prolonged exposure and/or physical activity
	39.4–51.1	Danger	Heat cramps or heat exhaustion likely, and heat stroke possible with prolonged exposure and/or physical activity
	Above 51.1	Extreme danger	Heat stroke highly likely

Previous literature made use of the HI to assess heat as a hazard in the Caribbean region (Ramirez‐Beltran *et al*., 2017; Angeles‐Malaspina *et al*., 2018). For comparison reasons, the present study considers the HI too. Unlike the UTCI, the HI is valid only in the upper range of thermal exposure, that is, heat. The HI classification and corresponding physiological responses are reported in Table 1. The HI is calculated from 2 m air temperature and relative humidity using the regression equation of Rothfusz (NOAA, 2014),

(1) HI=−42.379+2.04901523*T+10.14333127*RH−0.22475541*T*RH−0.00683783*T*T−0.05481717*RH*RH+0.00122874*T*T*RH+0.00085282*T*RH*RH−0.00000199*T*T*RH*RH,

where *T* is temperature in degrees Fahrenheit (°F) and RH is relative humidity in percent. Its output is HI, also known as apparent temperature, expressed in °F. If relative humidity is less than 13% and air temperature is between 80 and 112°F (26.7 and 44.4°C), then the following is subtracted from Equation (1):

(2) $$\text{Adjustment}=\left[\frac{13-\mathrm{RH}}{4}\right]*\sqrt{\frac{\left[17-\left|\left(T-95\right)\right|\right]}{17}}.$$

If relative humidity is greater than 85% and air temperature is between 80 and 87°F (26.7 and 30.6°C), then the following is added to Equation (1):

(3) $$\text{Adjustment}=\left[\frac{\mathrm{RH}-85}{10}\right]*\left[\frac{87-T}{5}\right].$$

If conditions of air temperature and relative humidity warrant a HI value below 80°F, a simpler formula is applied to calculate values consistent with Steadman's results:

(4) $$\mathrm{HI}=0.5*\left\{T+61.0+\left[\left(T-68.0\right)*1.2\right]+\left(\mathrm{RH}*0.094\right)\right\}.$$

Finally, HI is converted to °C.

### 2.3 Reanalysis datasets

The source of UTCI data is ERA5‐HEAT, a quality‐controlled reanalysis consisting of global gridded maps of the index from 1979 to present (Di Napoli *et al*., 2021a). In ERA5‐HEAT, the UTCI is derived using the *operational* procedure with climate variables from the ERA5 reanalysis, which is produced by the European Centre for Medium‐Range Weather Forecasts (ECMWF) within the Copernicus Climate Change Service (Hersbach *et al*., 2020). UTCI reanalysis data were retrieved from ERA5‐HEAT via the Copernicus Climate Data Store (CDS, 2020).

As for the heat stress drivers, reanalysis data of mean radiant temperature (MRT) were also retrieved from ERA5‐HEAT as representative of the solar and thermal radiation irradiating, both directly and diffusely, a human subject placed in an outdoor environment (Di Napoli *et al*., 2020). ERA5 was used as the reanalysis data source for 2 m air temperature, 2 m dew point temperature, and 10 m wind speed (CDS, 2018). Both ERA5 and ERA5‐HEAT are publicly and freely available for download from the Copernicus Climate Data Store (CDS, 2018; 2020).

Reanalysis data were used to derive climate variables not directly provided by reanalysis datasets. Relative humidity, for instance, was computed from 2 m air temperature and 2 m dew point temperature via the Clausius–Clapeyron relation (Alduchov and Eskridge, 1996). The HI was calculated from 2 m air temperature and relative humidity using Equations ((1), (2), (3), (4)).

All reanalysis data covered a period of 41 years (1980–2020) at 1‐hr time step, were cropped to the study area and, at each time step, consisted of 7,956 grid cells at 0.25° spatial resolution (31 km circa).

### 2.4 Statistical methods

The following statistical methods were used to investigate heat stress conditions and associated trends in the Caribbean region.

#### 2.4.1 Climatologies

The thermal bioclimate of the Caribbean, that is, the long‐term occurrence of heat stress in the region, was obtained from the daily minima, maxima, means of hourly UTCI reanalysis data for the 1980–2019 historical period. These daily statistics were chosen based on their relevance in a human health perspective. Specifically, daily maxima are considered representative of the daytime heat individuals might be exposed to, with potential direct detrimental effects (e.g., heatstroke); daily minima are considered representative of night‐time conditions which, for example, during heatwaves may deteriorate sleep quality and prevent individuals from recovering from daytime heat exposure; daily means are considered representative of the average heat experienced within a full day (McGregor *et al*., 2015). These statistics have all been associated to negative health impacts as well as implemented in operational heat health watch warning systems (Kotharkar and Ghosh, 2022). To address the objectives of the present study, UTCI daily minima, maxima, means were first computed for each grid cell during the hours from 2300 to 2200 UTC (Coordinated Universal Time) and then averaged across months and seasons to obtain corresponding climatologies of heat stress and in agreement to previous literature on the topic (e.g., Ramirez‐Beltran *et al*., 2017; Angeles‐Malaspina *et al*., 2018).

Seasons are defined as February–April (FMA), May–July (MJJ), August–October (ASO), and November–January (NDJ). This follows the bimodal rainfall distribution observed in the Caribbean region, that is, an early (MJJ) and late (ASO) rainy season interrupted by a dry spell in July (*mid‐summer drought*; Stephenson *et al*., 2014). Dry seasons, NDJ and FMA, correspond to the time of the year where the lowest rainfall intensity events are observed (Giannini *et al*., 2000; Chen and Taylor, 2002). The thermal bioclimate of FMA, MJJ, ASO, and NDJ was obtained by averaging UTCI daily minima, maxima, means across each season in the 1980–2019 period. This produced a set of pan‐Caribbean UTCI maps of season‐specific heat stress.

Monthly UTCI averages were computed over the same period to investigate intraseasonal changes in the UTCI climatology. They were also aggregated for geographical zones, that is, the entire study area as well as the regions corresponding to the Greater Antilles and the Lesser Antilles (Figure 1). This allowed for comparison between region‐wide trends and trends derived subregionally. The same methodology was applied to derive climatologies for heat stress drivers and the HI.

Finally, the monthly frequency of UTCI stress categories and HI danger categories was calculated. This offers insights on the local bioclimate in terms of the number of occurrences of heat stress/danger conditions to which local population are exposed across the year.

#### 2.4.2 Trends

Linear trends for the UTCI were computed in order to check for temporal changes in heat stress. The Sen's slope estimator was used to evaluate the rate of changes and the Mann–Kendall nonparametric statistical test was used to evaluate the significance level of the trends (Mann, 1945; Sen, 1968; Kendall, 1975). The combination of Mann–Kendall test and Sen's estimator has been successfully used in multiple climate trends studies focussed on the Caribbean (Aguilar *et al*., 2005; Beharry *et al*., 2015; Angeles‐Malaspina *et al*., 2018; Cavazos *et al*., 2019; Mohan *et al*., 2020). UTCI trends were calculated from averaged seasonal values for each year for the 1980–2019 historical period. The year‐by‐year change in heat stress, represented by Sen's slope coefficients, was then multiplied by 10 so to be represented as a decade‐by‐decade variation. Linear trends were calculated at each grid cell, thus allowing for investigation in UTCI trends' spatial patterns. The same linear trend analysis was applied to heat stress drivers and the HI.

Pearson correlations between mean yearly averages of the UTCI and mean yearly averages of 2 m air temperature, relative humidity, 10 m wind speed, and MRT were computed. This was done to highlight, both spatially and temporally, the relationships between the UTCI and each of its drivers during the 1980–2019 historical period. The strength of the correlation, represented by the absolute value of the Pearson correlations *r*, follows the classification by (Evans, 1996): 0.00–0.19 is “very weak”; 0.20–0.39 is “weak”; 0.40–0.59 is “moderate”; 0.60–0.79 is “strong” and 0.80–1.0 is “very strong.”

Geographical areas where trends are not statistically significant (*p* ≥ .05, where *p* is the probability level) were excluded from the analysis.

Additionally, anomalies by decades were computed for year‐ and season‐averaged UTCI and HI daily means with respect to the 1981–2010 climatological baseline.

#### 2.4.3 Multi‐index heat information

As described in section 2.2, UTCI values are categorized in terms of thermal stress, that is, of the physiological responses put in place by the human body to maintain its core temperature within the range of optimal physiological performance (McGregor and Vanos, 2018). The HI classification reflects the onset of more dangerous heat disorders under prolonged exposure and/or physical activity in the heat (NOAA NWS, 2021b). The co‐occurrence of UTCI thermal stress categories for different HI danger categories was assessed in the present study. This allowed for investigating the UTCI and the HI as heat metrics providing complementary information on the effects of heat on human health. The joint occurrence of UTCI stress categories and HI danger categories was determined at each grid cell of the seasonal averages (daily maxima) across the 1980–2019 historical period. Outputs were later aggregated for geographical zones. The analysis focuses on daily maxima to account for conditions of highest exposure to heat.

#### 2.4.4 A case study: The 2020 heat season

Two heatwaves affected several Caribbean states in April and September 2020. They are here analysed as a case study of heat extremes in the region. The monthly averaged values achieved by 2 m air temperature, the UTCI, and the HI were computed for the months when the heatwave occurred. The exceptionality of the events was assessed in terms of the anomalies of those variables to the 1980–2019 historical period. For the UTCI and HI, anomalies were expressed in differences of values in °C or of stress/danger categories. For the 2 m air temperature, which is not classified in categories, anomalies were expressed with respect to the exceedance of climatologically defined percentile thresholds, namely the 95th and 99.5th percentiles. The choice of these percentiles is justified by their linkages to human health in heat‐related impacts studies (Di Napoli *et al*., 2019).

## 3 RESULTS

### 3.1 Heat stress climatology of the Caribbean

**FIGURE 2:**
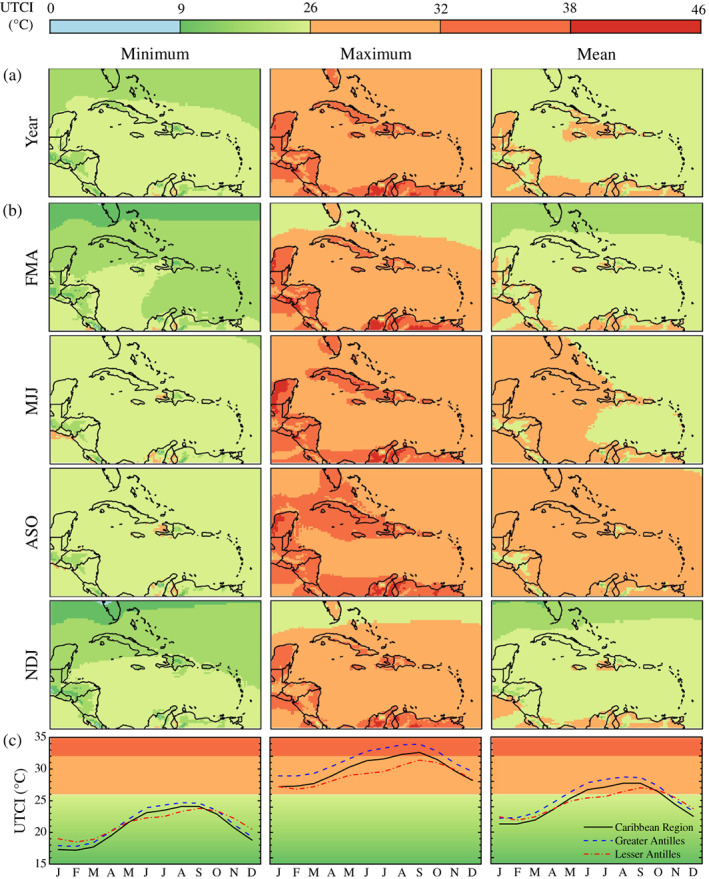
Yearly (a), seasonal (b), and monthly (c) UTCI climatology for the 1980–2019 historical period. Background colours refer to the UTCI stress category scale at the top of the figure [Colour figure can be viewed at wileyonlinelibrary.com]

The yearly climatology derived from 1980 to 2019 ERA5‐HEAT data show that heat stress is moderate, strong, and very strong in UTCI maxima, whereas UTCI minima are characterized by no thermal stress and UTCI means are associated with both no thermal stress and moderate heat stress (Figure 2a).

Season‐averaged UTCI maps confirm that UTCI minima are mostly associated to no thermal stress throughout the year (Figure 2b). UTCI maxima reveal extended moderate heat stress across the region with levels of strong and very strong heat stress achieved in islands such as Cuba, Hispaniola, Puerto Rico, and Bahamas as well as the northern part of South America and the Yucatán peninsula. This translates into UTCI means associated to prevalent conditions of no thermal stress in NDJ and FMA, of moderate heat stress in ASO, and between absent and moderate heat stress in MJJ. The lowest UTCI values occur at latitudes above 25°N circa, concurrently with the winter and spring seasons in the Northern Hemisphere. The highest UTCI values are widespread in the rainy season, ASO specifically, and indicate this as the heat stress season for the Caribbean.

The monthly climatology of the UTCI maximum for the average of the entire study area provides further insight into the heat stress occurrence throughout the year (Figure 2c). The UTCI monthly maximum starts ascending in March, it plateaus in June, it increases again reaching the highest value in September, then it descends until December and reaches a second plateau from January to March (or minimum in February), completing the annual cycle. The pattern is similar to the climatologies of UTCI minima and mean values. From June to September, the Greater Antilles exhibit higher UTCI values than both the average of the Caribbean region and the Lesser Antilles (33.9°C against 32.6 and 31.4°C, respectively, in September). For the Lesser Antilles, the UTCI increase between June and September is greatest (1.5–2.1°C against 0.7–1.1 and 1–1.3°C for the entire region and the Greater Antilles, respectively), but the UTCI varies the less between September and February (2.1–5.3°C against 5–6.9°C for the Caribbean region and the Greater Antilles).

The thermal bioclimate of the Caribbean is also reflected by the monthly frequencies of occurrence of different heat stress categories (Figure S1, Supporting Information). Conditions of no thermal stress prevail for UTCI minima throughout the year with moderate heat stress achieved in less than 10% of the days between May and October (peak in September) in the Greater and Lesser Antilles. UTCI maxima are predominantly characterized by heat stress. In the whole region, moderate heat stress occurs most frequently (68–100% of days) from October to June whereas strong heat stress is achieved in more than 71% of days in August and September. In the Greater Antilles, the high frequency (74–99%) of days with UTCI maxima in the strong heat stress category is observed over a longer period, from June to October. In the Lesser Antilles, moderate heat stress prevails in the UTCI maxima throughout the year and strong heat stress occurs with the highest frequency (30%) in September. As a consequence, moderate heat stress in UTCI means represents the dominant condition between July and September in the Caribbean region (77–97% of days), between May and October in the Greater Antilles (63–100% of days) and between August and October in the Lesser Antilles (70–82% of days).

### 3.2 Climatology of heat stress drivers

**FIGURE 3:**
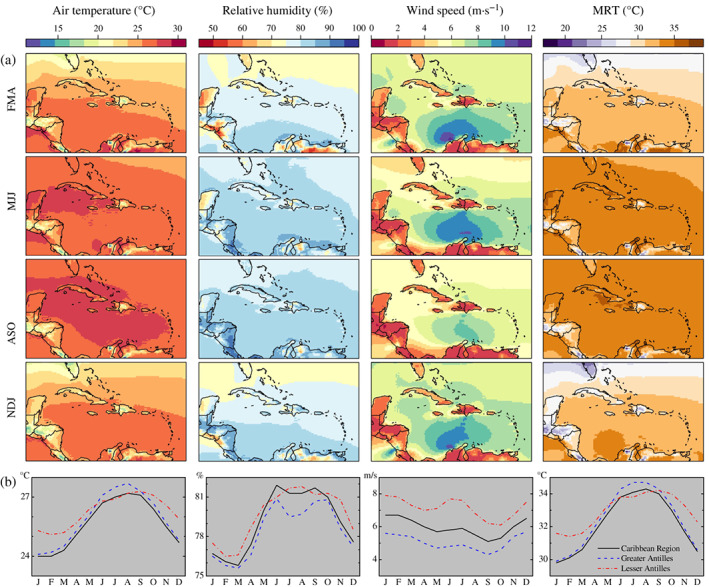
Mean climatology of UTCI drivers by seasons (a) and months (b) over the 1980–2019 historical period [Colour figure can be viewed at wileyonlinelibrary.com]

Figure 3 provides a pan‐Caribbean overview of the mean climatology of 2 m air temperature, relative humidity, 10 m wind speed, and MRT as derived from ERA5 and ERA5‐HEAT data for the 1980–2019 historical period.

Season‐averaged maps (Figure 3a) show that air temperature and MRT are at their maximum in ASO and minimum in NDJ, reflecting the amount of solar insolation the region receives because of its proximity to Earth's equator. Relative humidity is at its highest in MJJ and ASO, in agreement with the bimodal rainfall distribution. The strongest wind speeds for the study area are observed during FMA and are collocated with the Caribbean Low‐Level Jet that peaks during the same season (Whyte *et al*., 2008). The lowest wind speeds are instead achieved in ASO. Together, season‐averaged maps of heat stress drivers highlight ASO as the season characterized by general conditions of higher air temperatures, humidity, and radiation, and lower wind speeds.

Monthly climatologies, aggregated across the Caribbean region, further support the heat characterization of the ASO season (Figure 3b). They show a unimodal distribution for 2 m air temperature and MRT, with the two climate variables reaching a maximum in August (27.2 and 34.3°C) and a minimum in January (24 and 29.8°C). Relative humidity exhibits a bimodal distribution that increases in March, reaches a first peak in June (82%), decreases and plateaus in July–August, increases again to a second peak in September (82%) and then decreases until March (76%), completing the annual cycle. Wind speeds attain a minimum in September (5.1 m·s^−1^), a primary maximum in January (6.7 m·s^−1^), and a secondary maximum in July (5.9 m·s^−1^). It is worth noting that the secondary maximum coincides with the occurrence of the Caribbean mid‐summer drought and wind speeds are higher during the dry season (6–6.7 m·s^−1^) than the rainy season.

At the subregional level, the climatologies for 2 m air temperature, relative humidity, 10 m wind speed, and MRT are similar in pattern between the Greater Antilles and the Caribbean‐averaged ones. The unimodal distribution of air temperature (resp. MRT) reaches a minimum of 24.1°C (resp. 29.9°C) in January and a maximum of 27.7°C (resp. 34.7°C) in July–August. These values are above the Caribbean‐averaged ones. With regards to relative humidity and wind speed, the Greater Antilles are drier (80.9% as maximum value) and less windy (4.3–5.7 m·s^−1^) than the region overall.

The climatology of each heat stress driver in the Lesser Antilles displays region‐specific distributions. Air temperature there reaches its maximum in September (27.3°C), 1 month later than the Greater Antilles. It is also characterized by a higher minimum (25.1°C in February). MRT follows a similar distribution with maximum and minimum values equal to 34.2 and 31.4°C in September and February, respectively. It follows that the change in air temperature and MRT (2.2 and 2.8°C) during the year is less marked across the Lesser Antilles than the Greater Antilles (3.6 and 4.8°C). As for relative humidity, a primary maximum is observed during July–August (81.7–81.8%), followed first by a small reduction and then a slight increase in October (81.3%), after which it descends completing the annual cycle in February–March. Wind speeds follow the same distribution as the Caribbean‐ and the Greater Antilles‐averaged ones, with higher values throughout the year (6.1–7.9 m·s^−1^).

### 3.3 Trends in heat stress

**FIGURE 4:**
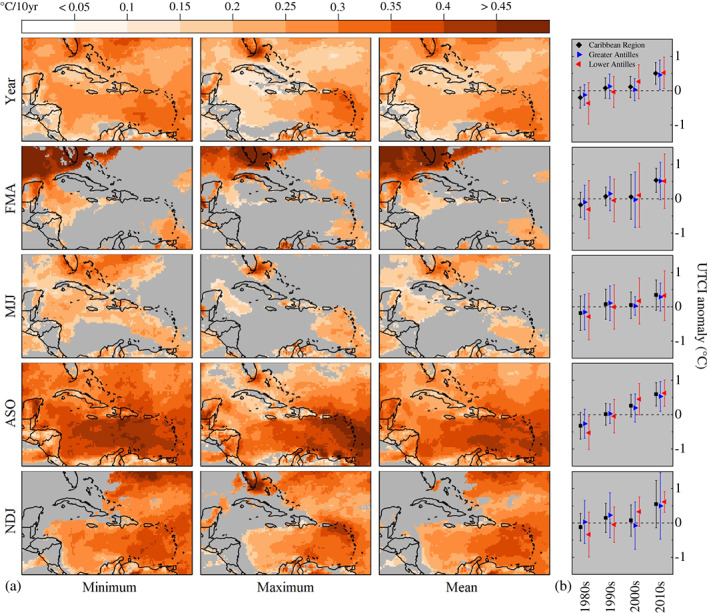
Spatiotemporal trends of the UTCI in the Caribbean region. (a) Decadal trends, as represented by Sen's slope coefficients, in minimum, maximum and mean UTCI for whole years and by seasons over the 1980–2019 period. Grey areas indicate grid cells where trends are not statistically significant according to the Mann–Kendall test (*p* ≥ .05). (b) Decadal anomalies of mean UTCI to the 1981–2010 climatological baseline by regions. Error bars represent one standard deviation [Colour figure can be viewed at wileyonlinelibrary.com]

Linear trends of the UTCI are calculated for the Caribbean region (Figure 4a), and an increasing trend for the year‐round heat stress is observed. During the 1980–2019 historical period, UTCI minima have increased more than 0.2°C·decade^−1^ in 81% of the Caribbean. UTCI maxima have increased more than 0.2°C·decade^−1^ in 49% of the region with increases above 0.3°C·decade^−1^ being observed in areas such as southern Florida and the Lesser Antilles. Furthermore, heat stress trends differ according to the season. Statistically significant increases in the UTCI (minimum, mean, and maximum) are found in ASO for the whole Caribbean region, with the Lesser Antilles experiencing rates exceeding 0.45°C·decade^−1^. The same rate is observed in southern Florida during the Caribbean dry season, specifically FMA. It is worth noting that the Lesser Antilles show a statistically significant increase in all seasons and statistics (minima, maxima, and means).

A further insight into how heat stress has changed in the Caribbean region across 40 years is provided by UTCI anomalies relative to the 1981–2010 climatology baseline (Figure 4b). In the 2010–2019 decade, yearly data indicate that region‐averaged UTCI was 0.51°C higher than the baseline and 0.71°C higher than the 1980–1989 decade. In the 1990–1999 and 2000–2009 decades, the UTCI was closest to the baseline although the Lesser Antilles show an increasing trend throughout the entire baseline period. These findings can also be observed in season data, namely in FMA, MJJ, and NDJ. For ASO, there is not increase hiatus during the 1990–2009 period and the UTCI increases from the 1980–1989 decade to the 2010–2019 throughout. Such a continuous increase over time is observed for the Lesser Antilles, the Greater Antilles, and the entire Caribbean region.

### 3.4 Trends in heat stress drivers

**FIGURE 5:**
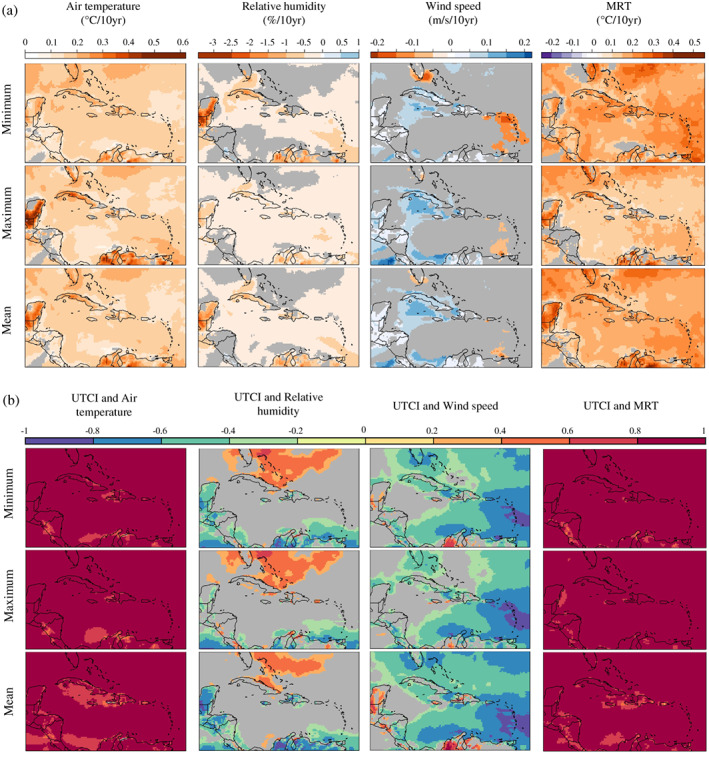
Spatiotemporal trends of heat stress drivers in the Caribbean region. (a) Decadal trends, as represented by Sen's slope coefficients, in minimum, maximum and mean yearly averages of 2 m air temperature, relative humidity, 10 m wind speed and MRT over the 1980–2019 historical period. Grey areas indicate grid cells where trends are not statistically significant according to the Mann–Kendall test (*p* ≥ .05). (b) Pearson correlation between mean yearly averages of the UTCI and its drivers over the 1980–2019 historical period. Geographical areas with not statistically significant correlation (*p* ≥ .05) are shown in grey [Colour figure can be viewed at wileyonlinelibrary.com]

Figure 5a presents linear trends for each of the UTCI heat stress drivers. Since 1980 the Caribbean region has been warming by up to 0.18°C·decade^−1^, with some areas such as the northwestern Cuba, the Yucatán peninsula and the northern part of South America experiencing increases in air temperature maxima above 0.25°C·decade^−1^. MRT has also been increasing in the region, with trends up to 0.24°C·decade^−1^ in the minima in the Lesser Antilles. The region has become drier with minima of relative humidity decreasing up to 0.53%·decade^−1^. As for the wind speed, this has been increasing in some parts of the Caribbean region, such as Cuba, Jamaica, and Hispaniola, and decreasing in others, for example, the Lesser Antilles and southern Florida. This is particularly evident in wind speed minima. Trends by season indicate ASO as the season when air temperature and MRT have increased at the highest rates across most of the Caribbean, and wind speed has decreased more than 0.15 m·s^−1^·decade^−1^ in the Lesser Antilles; similar rates and trends are observed in southern Florida during FMA (Figures S2–S5).

Figure 5b illustrates the correlation between mean yearly averages of the UTCI and mean yearly averages of each of its drivers. The UTCI and 2 m air temperature (resp. MRT) were found to be strongly positively correlated during the 1980–2019 historical period, with an average correlation coefficient *r* of 0.91 (resp. 0.92) in more than 89% (resp. 94%) of the Caribbean region. On the contrary, the UTCI and wind speed were found to be negatively correlated. The magnitude of the correlation is strongly negative for the Lesser Antilles (*r* = −.77 ± .01) and moderately negative for the Greater Antilles (*r* = −.45 ± .01). UTCI and relative humidity trends were found to be both positively and negatively correlated depending on the sub‐regional location. Weak to very strong positive correlations are found for the Greater Antilles, particularly the Bahamas and Puerto Rico, whereas weak to strong negative correlations are observed in the southern part of the Lesser Antilles.

### 3.5 Heat danger and heat stress

**FIGURE 6:**
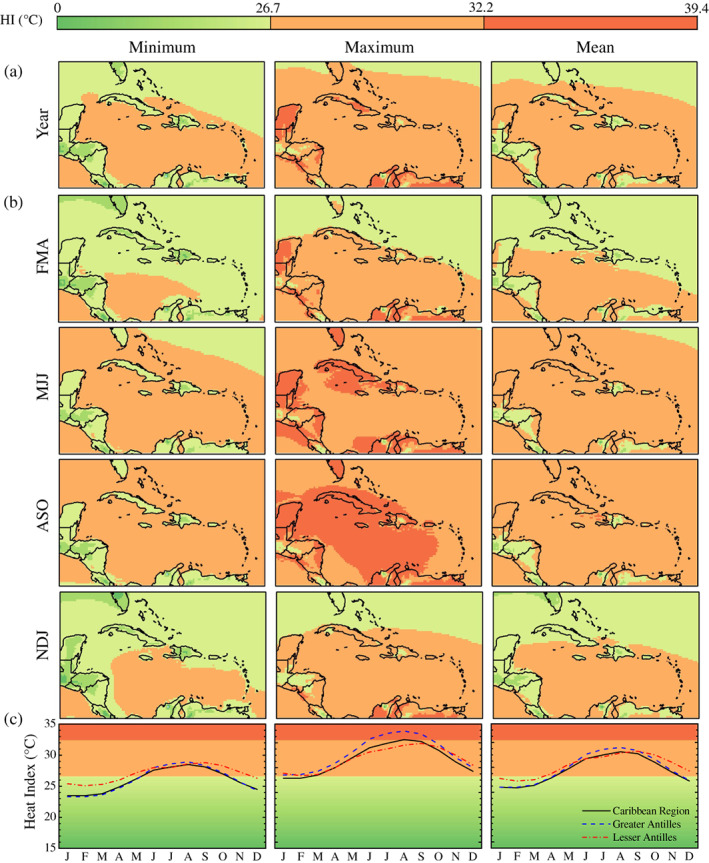
Yearly (a), seasonal (b), and monthly (c) heat index climatology for the 1980–2019 historical period. Background colours refer to the heat index danger category scale at the top of the figure [Colour figure can be viewed at wileyonlinelibrary.com]

HI climatology derived from ERA5 data for the 1980–2019 historical period shows yearly HI maxima mostly associated to caution and extreme caution, and it identifies ASO as the season when exposure to heat may be more detrimental (Figure 6a,b). In ASO daily maxima, an extreme caution area spans from the Greater Antilles to Florida, the Yucatán peninsula and part of the southern American Caribbean coastline, including the islands of Aruba and Curacao. HI values associated with caution dominate the rest of the region for both daily maxima and minima. Conditions of no heat‐related danger are mostly prevalent in NDJ and FMA. As for the monthly climatology, the HI follows a unimodal distribution that has a minimum in January–February and a maximum in August (Figure 6c). For most of the year the Greater Antilles experience higher maximum and mean HI values than both the entire averaged Caribbean region and the Lesser Antilles. On the contrary, the Lesser Antilles are exposed to higher minimum HI values than the rest of the region. Furthermore, the Lesser Antilles experience the maximum peak in the HI later in the year, in September.

Monthly frequencies of HI danger categories are consistent with above described monthly climatologies (Figure S6). Daily minima associated with the caution category occur most frequently between July and October across the Caribbean (66–100% of days) and the Greater Antilles (69–99% of days), and between May and November (70–98% of days) in the Lesser Antilles. In daily maxima, extreme caution is the most frequent condition in August and September across the Caribbean (56–68% of days) and between June and September (70–99% of days) in the Greater Antilles. On the other hand, days with HI maxima associated with caution dominate the climatology of the Lesser Antilles throughout the year, with extreme caution observed in no more than 42% of days. Caution is the most frequent category in daily means in at least 7 months of the year, in agreement with the HI monthly climatology, and reaches extreme levels in up to 16% (resp. 6%) of days in August (resp. September) in the Great Antilles (resp. the Lesser Antilles).

Positive trends are observed across the region for the HI during the 1980–2019 historical period (Figure S7). According to annual data, increases greater than 0.2°C·decade^−1^ occurred in all statistics (minima, maxima, and means) in more than 91% of the reanalysis grids that compose the Caribbean region, with the highest trends experienced in the southern part of the Lesser Antilles, the Yucatán Peninsula, and the northern part of South America. Seasonal data reveal trends with statistically significant increases greater than 0.45°C·decade^−1^ in 43% of the region during ASO, and in southern Florida during FMA. In terms of anomalies to the 1981–2010 climatology baseline, these were, in the decade 2010–2019 and for region‐averaged HI, between +0.5°C and +0.8°C with the latter achieved in ASO. In the Lesser Antilles, the HI has been continuously increasing and, in the late rainy seasons of 2010s, was +1.2°C higher than the 1980–1989 decade. On the contrary, in the Greater Antilles and at the region level, an increase hiatus can be observed in FMA, MJJ and NDJ during the 1990–1999 and 2000–2009 decades.

Table 2 shows the joint occurrence of UTCI stress categories and HI danger categories for daily maxima in ASO. When the exposure to heat is at its highest, conditions of “moderate heat stress” occur simultaneously to conditions of “caution” in 45.5% of the Caribbean region. Conditions of “strong heat stress” are associated to conditions of “extreme caution” in 20.6% of the region, immediately followed by the joint occurrence of “moderate heat stress” and “extreme caution” (12.4%). For the Greater Antilles, the co‐occurrence of “strong heat stress” and “extreme caution” dominates (74%) whereas for the Lesser Antilles conditions of “moderate heat stress” and “caution” are more prevalent (97.2%).

**TABLE 2:** Joint distribution in percentage of UTCI stress categories and HI danger categories across the whole Caribbean region during the 1980–2019 historical period for ASO maxima

HI/UTCI	No danger	Caution	Extreme caution	Danger	Extreme danger
No thermal stress	0	0	0	0	0
Moderate heat stress	0.8 [0.5; 0]	45.5 [7.9; 97.2]	19.2 [9.2; 0.2]	0	0
Strong heat stress	0.2 [0.1; 0]	12.4 [7.5; 1.5]	20.6 [74; 1.2]	0	0
Very strong heat stress	0	0	1.3 [0.9; 0]	0	0
Extreme heat stress	0	0	0	0	0

*Note*: Values in squared brackets refer to corresponding percentages across the Greater Antilles (left) and the Lesser Antilles (right).

### 3.6 Case study: The 2020 heat season

**FIGURE 7:**
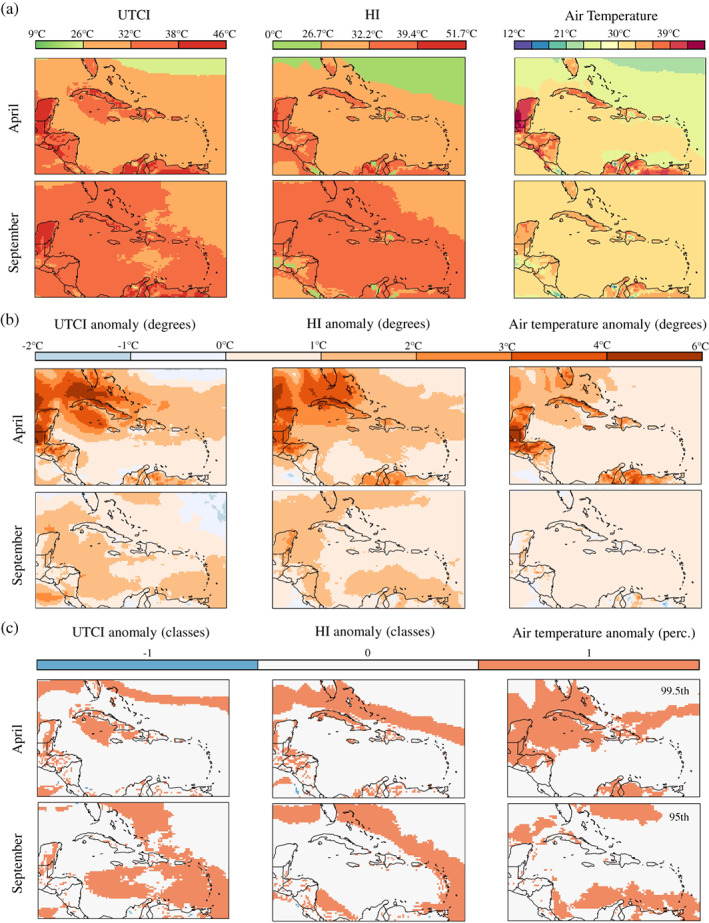
Bioclimate analysis of the heatwaves that affected the Caribbean region in April and September 2020. (a) Monthly means of daily maxima for the UTCI, HI and 2 m air temperature. (b) Anomalies to the 1980–2019 historical period in °C. (c) Anomalies to the 1980–2019 period in classes for the UTCI and HI, and with respect to climatological‐defined percentiles for air temperature [Colour figure can be viewed at wileyonlinelibrary.com]

Figure 7 illustrates the record‐breaking heat season of 2020 in terms of the UTCI, HI and 2 m air temperature, and of the months, April and September, when two heatwaves hit the Caribbean.

In April 2020 monthly averages of daily maxima highlight, for Cuba, air temperatures between 33 and 39°C as well as conditions of strong to very strong heat stress in the UTCI and of extreme caution in the HI. The exceptionality of the April 2020 heatwave is reflected in the anomalies to the 1980–2019 historical period. An area stretching from southern Florida to Yucatán through the Bahamas, Cuba, Jamaica and Cayman Islands, experienced higher‐than‐average air temperatures and heat with the UTCI and HI up to 6°C above their 1980–2019 values. Heat stress and heat danger were up to 1 category higher than expected from the period, and air temperatures exceeded the 99.5th percentile demonstrating the exceptionality of the event.

In September 2020 conditions associated to strong heat stress and extreme caution reached the Lesser Antilles. Here anomalies in UTCI and HI values were up to 2°C and, although more contained than the anomalies in April, they brought heat stress and heat danger categories up to 1 category with respect to the 1980–2019 historical period. Air temperatures exceeded 30°C and the 95th percentile in the southern part of the Lesser Antilles.

## 4 DISCUSSION

This study presents the first assessment of human heat stress in the Caribbean region. Using climate reanalysis datasets (ERA5 and ERA5‐HEAT), it explores heat stress bioclimatology and trends over the period from 1980 to 2019 from a spatiotemporal perspective.

Considering the Universal Thermal Climate Index (UTCI) as a representative measure for the heat stress endured by the human body when exposed to outdoor conditions, pan‐Caribbean bioclimatology maps of the UTCI denote August–September–October (ASO) as the season when conditions of heat stress up to very strong levels dominate the region, particularly the Greater Antilles, as well as the Yucatán peninsula and the Caribbean coast of South America. This is reflected in the frequency of days when heat stress is achieved. During ASO, the Greater and Lesser Antilles experience UTCI minima associated to moderate heat stress in up to 10% of days, and UTCI maxima in the strong heat stress category between 30 and 99% of days. The analysis of climate variables known to drive heat stress reveals the meteorological reason why the UTCI reaches the highest values during ASO. From March, 2 m air temperature, relative humidity, and mean radiant temperature (MRT) increase concurrently until June, when they plateau and remain at their maximum, with peaks in August and September. During the same months, 10 m wind speed decreases, reaching its lowest in September. From October, the joint decrease of 2 m air temperature, relative humidity, and MRT and increase of wind speed drives the descending of the UTCI which reaches a minimum in February. This finding reflects the thermophysiological nature of the UTCI. High temperatures, exposure to insolation, poor ventilation, and high levels of atmospheric moisture contribute synergistically to raise the heat content of the human body (McGregor and Vanos, 2018).

This study also identifies ASO as the season when the UTCI has been increasing the most since 1980. In the last decade (2010–2019) the Caribbean region has experienced the highest heat stress levels of the past 40 years, with the UTCI 0.51°C higher than the 1981–2010 climatology baseline. In a global context, such an increase is lower than that observed at higher latitudes, for example, in Europe (Di Napoli *et al*., 2018; Antonescu *et al*., 2021). Long‐term observations from Poland, however, indicate a decadal trend like the Caribbean one for mean UTCI values during the Northern Hemisphere's summer season (0.55 ± 0.10°C) (Kuchcik *et al*., 2021). This supports the importance of the subcontinental/subregional analysis here presented. It is worth noting that high rates in the increase of the UTCI (0.45°C·decade^−1^) are observed in southern Florida during the Caribbean dry season, specifically FMA. This agrees with the decreasing trends in cold stress observed at higher latitudes in the winter/early spring season (Błażejczyk and Twardosz, 2010; Varentsov *et al*., 2020; Kuchcik *et al*., 2021; Vinogradova, 2021).

Increasing exposure to extreme heat has been recently identified as a slow onset event with negative implications worldwide but particularly in tropical regions (Oppermann *et al*., 2021). The bioclimatology analysis here presented (UTCI maxima; Figure 2) reveals that people living in the Caribbean chronically experience moderate to very strong heat stress. Because of this, they may require less warming to achieve lethal heat stress levels and may incur in heat‐related risks despite being used to high level of heat stress. The upward trend of the UTCI also calls for the further investigation of sudden onset heat events, namely heatwaves, during ASO as it has been shown that heatwaves are more likely to occur during the rainy season and less likely to take place during the dry season (Ramirez‐Beltran *et al*., 2017). Furthermore, ASO falls in the hurricane season, and heatwaves have been observed occurring immediately after major hurricanes (Matthews *et al*., 2019; NOAA NWS, 2021a). Compound heatwave‐hurricane events may expose local population to an exacerbated heat risk as hurricanes are responsible for major disruptions in essential services such as electricity and potable water that are critical for survivability during heat extremes (Méndez‐Lázaro *et al*., 2021). Heatwaves as compound hazards should therefore also be investigated in the Caribbean region. This will assess whether heat and its extremes can be a limitation to sustainable development in the Caribbean as is cold stress in high‐latitude countries (Varentsov *et al*., 2020; Vinogradova, 2021). Considering regional economic grouping such CARICOM (Caribbean Community and Common Market; Jones *et al*., 2016b) could help towards this as well as extend the present analysis to countries in the southern Caribbean (Guyana and Suriname).

With regards to heat stress drivers, the Caribbean region has become warmer and drier. These findings, based on reanalysis data, agree with previous studies based on observations from meteorological stations which also observed statistically significant increases in air temperature during the wet season in Barbados and Tobago (Singh, 1997; Peterson *et al*., 2002; Aguilar *et al*., 2005; Pérez and Jury, 2012; Stephenson *et al*., 2014; Beharry *et al*., 2015; Dookie *et al*., 2018; Cavazos *et al*., 2019; Mohan *et al*., 2020).

Decadal trends in MRT indicate that the total radiation (solar and thermal) irradiating a human subject placed in an outdoor environment has been increasing. An upward trend has been found for solar radiation in Puerto Rico (Jury, 2015). As for the thermal component of MRT, which refers to radiation emitted and absorbed by land surface, clouds, and other components of the atmosphere–land system, cloud cover data have shown that cloudiness has been increasing in the Caribbean region, especially at night, thus blocking more thermal radiation from land surface and contributing to a net heating effect (Singh, 1997; Yu *et al*., 2017).

Spatial patterns and decadal trends of wind speed are in agreement with previous findings (Chadee and Clarke, 2014). The increase observed in the 1980–2019 historical period across the western Caribbean Sea is expected to continue in the future (Angeles *et al*., 2010; Costoya *et al*., 2019). To the authors' knowledge, the decrease in wind speed minima observed for the Lesser Antilles has not yet been documented in the literature. To investigate this, future studies can explore wind speed records from weather stations and compare them against data from ERA5 reanalysis. This would expand and update existing research which reports a good agreement between weather station observations and data from ECMWF previous reanalyses, namely ERA‐40 and ERA‐Interim (Jury, 2012; Pérez and Jury, 2012; Chadee and Clarke, 2014). It is acknowledged that although reanalysis datasets have many advantages they may also suffer from biases due to (a) the inherent uncertainties of the numerical weather prediction model used to generate them, and (b) their nature as a collection of grid cells, that is, values averaged over an area (Parker, 2016). Biases can be identified by comparing the reanalysis against in situ measurements or observational/satellite bias‐corrected datasets. Applied to UTCI input variables, the comparison would reveal any bias and assess how this may impact the UTCI in turn. This validation approach, which can be explored in future studies, would allow ERA5‐HEAT to be corrected where needed, thus improving the skill of potential heat stress forecasts across the region. This approach has already proved successful for UTCI‐based forecasting systems currently operational in Europe (Di Napoli *et al*., 2021b).

In this study, reanalysis data are aggregated by geographical zones—the entire Caribbean, the Greater Antilles, and the Lesser Antilles—to allow for comparison between region‐wide and subregional derived trends. All three geographical zones include both grid cells on water bodies, such as the sea, and grid cells on land. As for the latter, they may correspond to coastal or hinterland areas according to the geographical location and/or size of each island. Furthermore, some reanalysis grid cells over water may not resolve the land cover from subgrid scale size Caribbean islands like Barbuda or Cayman Island. This may influence how heat metrics and heat stress drivers over islands are represented in the reanalysis. Because of this, the representativeness of ERA5‐HEAT in marine environments, outside populated islands, may be worth investigating in future studies.

The spatiotemporal correlation between heat stress and heat stress drivers has been assessed via the Pearson correlation coefficient. During the 1980–2019 historical period the UTCI is found to be negatively correlated with wind speed and positively correlated with MRT and 2 m air temperature (Pappenberger *et al*. (2015) and Zare *et al*. (2018) have previously shown the UTCI shows some linear dependencies on air temperature). This suggests the upward trend in heat stress might be driven by increases in air temperature and radiation and decreases in wind speed. The latter is particularly evident in the Lesser Antilles. The correlation between the UTCI and relative humidity is both positive and negative, albeit not statistically significant in most of the Caribbean region. It is worth noting that the correlation is positive in areas where relative humidity has no significant decadal trend. Future research could address this topic by investigating trends in relative humidity across the Caribbean and their connection to heat stress in the region.

Alongside the UTCI, this study investigates heat‐induced danger as expressed by the HI. The 1980–2019 climatology of the HI, here computed from ERA5 reanalysis data, agrees with the spatiotemporal distribution of the HI reported in current literature (Ramirez‐Beltran *et al*., 2017; Angeles‐Malaspina *et al*., 2018). To complement that, the monthly frequency of heat danger categories is here provided and reveals dominant conditions of caution for the Caribbean, with HI maxima associated to extreme caution in up to 42 and 99% of days in the Lesser and Greater Antilles, respectively. It is also found, for the first time, that the UTCI and the HI provide complementary information on the effects of heat on human health. Conditions associated to high UTCI values occur simultaneously to conditions characterized by high HI levels. This suggests that dangerous heat disorders occur when physiological responses to heat are most stressful.

In the 2010–2019 decade, a positive HI change (+1.2°C) is found with respect to the 1981–2010 climatology baseline. Trends greater than 0.45°C·decade^−1^ are observed during ASO across most of the Caribbean basin, with the southern Lesser Antilles experiencing statistically significant increases throughout the year. These findings are in agreement with previous literature (Lee and Brenner, 2015). With regards to the processes and patterns responsible for HI trends, HI intensification in the Caribbean has been shown to be driven by changes in sea level pressure, sinking dry air enhancement, as well as warm advection strengthening and weaker cold advection (Angeles‐Malaspina *et al*., 2018). These processes were found to influence humidity and wind speed patterns, especially during the Caribbean wet season (Angeles *et al*., 2010; Campbell *et al*., 2011), and correlations between air temperature variability and the Atlantic multidecadal oscillation (AMO) signal of the North Atlantic surface sea temperatures were demonstrated (Stephenson *et al*., 2014). As for the UTCI, evidence on the association between UTCI trends and circulation patterns is currently missing for the Caribbean. Preliminary studies unveiling the role of the North Atlantic Oscillation (NAO) on the variability of the UTCI in Eastern Europe (Głogowski *et al*., 2020) call for similar investigations at lower latitudes.

During the heatwaves that affected the Caribbean region in 2020, the UTCI and HI raised up to 1 category higher than their historical averages. This exposed local populations to conditions of heat stress and danger they might not be physiologically used to, with potential consequences to their health. The importance of assessing the links between extreme heat episodes and mortality has recently been demonstrated for San Juan, Puerto Rico, and contextualized in terms of local vulnerability (Méndez‐Lázaro *et al*., 2015; 2018a; 2018b). Future studies can investigate the effects of heat on public health across different Caribbean nations. Complementary to that, research efforts could focus on characterizing the years when heat stress anomalies to a climatological baseline have been observed or heatwaves have been reported and on identifying the corresponding driving mechanisms. Years with the highest incidence of HI‐defined extreme events, for instance, have been found to coincide with the cool phase of the El Niño–Southern Oscillation (ENSO; Ramirez‐Beltran *et al*., 2017).

Bringing together health and environmental information would provide local decision makers with evidence on how to address and mitigate heat‐related health hazards, also via integrated risk monitoring and early warning systems (WHO, 2018). To the authors' knowledge, Puerto Rico and Saint Lucia are the only Caribbean nations with an operational early warning system and an active response plan for extreme heat events, respectively (Government of Saint Lucia, 2006; NOAA NWS, 2016). The increase in heat stress observed across the whole Caribbean calls for the establishment of response plans in more nations. Furthermore, heat stress information as delivered by the UTCI could help strengthen local meteo‐climate services (Trotman *et al*., 2018). A UTCI‐based forecasting system, for example, could provide an added value to the experimental seasonal heat outlook that is currently run by the Caribbean Institute for Meteorology and Hydrology (CIMH) and complement its surface air temperature‐based forecasts.

## 5 CONCLUSIONS

Exposure to heat stress based on the UTCI has been determined and characterized in trends, drivers, and extremes across the Caribbean region for the first time.

Using 40 years of meteorological data from the ECMWF ERA5‐HEAT reanalysis, the Caribbean bioclimate has been investigated via UTCI maps computed for the dry (NDJ, FMA) and rainy seasons (MJJ, ASO). ASO was identified as the season when heat stress is the highest, most frequent, and widespread, particularly in the maxima. Strong to very strong heat stress is experienced in the Greater Antilles, the Yucatán peninsula, southern Florida, and the southern American Caribbean coastline. Besides a spatial variation across the Caribbean, heat stress has been witnessing a time variation too. From 1980 to 2019, the UTCI has increased more than 0.2°C·decade^−1^ in almost half of the region, reaching marked above‐climatology values especially in the last 10 years. The ASO season is characterized by the strongest upwards trends, with rates greater than 0.45°C·decade^−1^ in southern Florida and the Lesser Antilles.

Data from ECMWF ERA5 and ERA5‐HEAT reanalyses reveal that the Caribbean region has become warmer and drier, and it has been receiving increasing radiation. Being temperature, relative humidity, radiation, and wind speed, the drivers of human heat stress, a spatiotemporal correlation between the UTCI and such drivers is here investigated and found to be diverse. During the 1980–2019 period heat stress has increased as air temperature and radiation have increased, and wind speed has decreased. The latter is particularly evident in the Lesser Antilles and during ASO. Given the spatial dominance of the marine environment in the subregion, future studies could evaluate reanalysis data against station data in the Caribbean. The correlation between the UTCI and relative humidity is complex exhibiting a mix of positive and negative correlations, which cannot be easily explained and requires for further investigation.

The present study is also the first to provide a multi‐index heat assessment of the Caribbean region. Conditions of heat danger, as represented by the HI, have intensified since 1980 (+1.2°C) and are found to occur simultaneously to conditions of heat stress, as indicated by the UTCI. This supports the idea that the more physiological responses the human body must put in place to contrast an extremely hot environment, the more dangerous heat disorders it may develop. The exceptional 2020 heat season, here investigated as a case study, exposed local population to above‐average heat stress and danger.

These findings demonstrate that the Caribbean region is witnessing a gradual intensification of heat stress on top of rapid onset extreme heat events. Region‐wide maps of heat stress and UTCI trends provide an information platform that highlights when and where preparedness and response plans to heat are needed. Further studies that integrate heat stress data with data on social sectors such as public health could help quantify the impacts of heat in the Caribbean.

## FUNDING INFORMATION

Royal Society, FOS\R1\191010; Wellcome Trust, 209734/Z/17/Z.

## CONFLICT OF INTEREST

The authors declare no potential conflict of interest.

## Supporting information


**Figure S1** Monthly frequency of UTCI (Universal Thermal Climate Index) categories for the Caribbean region (a), the Greater Antilles (b), and the Lesser Antilles (c). The frequency refers to the percentage of days when daily minima, maxima and means fall in indicated UTCI categories over the 1980–2019 historical period.
**Figure S2** Spatiotemporal trends of 2 m air temperature (minimum, maximum, and mean) in the Caribbean region for the 1980–2019 period. Trends are represented by Sen's slope coefficients and shown by seasons. Grey areas indicate grid cells where trends are not statistically significant according to the Mann–Kendall test (*p* ≥ .05).
**Figure S3** Spatiotemporal trends of relative humidity (minimum, maximum, and mean) in the Caribbean region for the 1980–2019 period. Trends are represented by Sen's slope coefficients and shown by seasons. Grey areas indicate grid cells where trends are not statistically significant according to the Mann–Kendall test (*p* ≥ .05).
**Figure S4** Spatiotemporal trends of 10 m wind speed (minimum, maximum, and mean) in the Caribbean region for the 1980–2019 period. Trends are represented by Sen's slope coefficients and shown by seasons. Grey areas indicate grid cells where trends are not statistically significant according to the Mann–Kendall test (*p* ≥ .05).
**Figure S5** Spatiotemporal trends of mean radiant temperature (minimum, maximum, and mean) in the Caribbean region for the 1980–2019 period. Trends are represented by Sen's slope coefficients and shown by seasons. Grey areas indicate grid cells where trends are not statistically significant according to the Mann–Kendall test (*p* ≥ .05).
**Figure S6** Monthly frequency of HI (heat index) categories for the Caribbean region (a), the Greater Antilles (b) and the Lesser Antilles (c). The frequency refers to the percentage of days when daily minima, maxima and means fall in indicated HI categories over the 1980–2019 historical period.
**Figure S7** Spatiotemporal trends of the heat index (HI) in the Caribbean region. (a) Decadal trends, as represented by Sen's slope coefficients, in minimum, maximum and mean HI for whole years and by seasons over the 1980–2019 period. Grey areas indicate grid cells where trends are not statistically significant according to the Mann–Kendall test (*p* ≥ .05). (b) Decadal anomalies of mean HI to the 1981–2010 climatological baseline by regions. Error bars represent one standard deviation.
