# Supplementary material for: Heat stress in the Caribbean: Climatology, drivers, and trends of human biometeorology indices
Source: Int J Climatol. 2022 Jul 15;43(1):405–25. doi: 10.1002/joc.7774 (PMC10084168; doi:10.1002/joc.7774)
Supplement: Supplementary file 1 — Figure S1 Monthly frequency of UTCI (Universal Thermal Climate Index) categories for the Caribbean region (a), the Greater Antilles (b), and the Lesser Antilles (c). The frequency refers to the percentage of days when daily minima, maxima and means fall in indicated UTCI categories over the 1980–2019 historical period. Figure S2 Spatiotemporal trends of 2 m air temperature (minimum, maximum, and mean) in the Caribbean region for the 1980–2019 period. Trends are represented by Sen's slope coefficients and shown by seasons. Grey areas indicate grid cells where trends are not statistically significant according to the Mann–Kendall test (p ≥ .05). Figure S3 Spatiotemporal trends of relative humidity (minimum, maximum, and mean) in the Caribbean region for the 1980–2019 period. Trends are represented by Sen's slope coefficients and shown by seasons. Grey areas indicate grid cells where trends are not statistically significant according to the Mann–Kendall test (p ≥ .05). Figure S4 Spatiotemporal trends of 10 m wind speed (minimum, maximum, and mean) in the Caribbean region for the 1980–2019 period. Trends are represented by Sen's slope coefficients and shown by seasons. Grey areas indicate grid cells where trends are not statistically significant according to the Mann–Kendall test (p ≥ .05). Figure S5 Spatiotemporal trends of mean radiant temperature (minimum, maximum, and mean) in the Caribbean region for the 1980–2019 period. Trends are represented by Sen's slope coefficients and shown by seasons. Grey areas indicate grid cells where trends are not statistically significant according to the Mann–Kendall test (p ≥ .05). Figure S6 Monthly frequency of HI (heat index) categories for the Caribbean region (a), the Greater Antilles (b) and the Lesser Antilles (c). The frequency refers to the percentage of days when daily minima, maxima and means fall in indicated HI categories over the 1980–2019 historical period. Figure S7 Spatiotemporal trends of the heat index (HI) in the [file JOC-43-405-s001.docx]

**Supporting Information**

**Heat stress in the Caribbean: climatology, drivers, and trends of human biometeorology indices**

Claudia Di Napoli*

School of Agriculture, Policy and Development, University of Reading, Reading, UK

Department of Geography and Environmental Science, University of Reading, Reading, UK
European Centre for Medium Range Weather Forecasts, Reading, UK
**Corresponding author* – c.dinapoli@reading.ac.uk

Theodore Allen

Caribbean Institute for Meteorology and Hydrology, St James, Barbados

Pablo A. Méndez-Lázaro

Environmental Health Department, Graduate School of Public Health, University of Puerto Rico, Medical Sciences Campus, San Juan, Puerto Rico

Florian Pappenberger

European Centre for Medium Range Weather Forecasts, Reading, UK


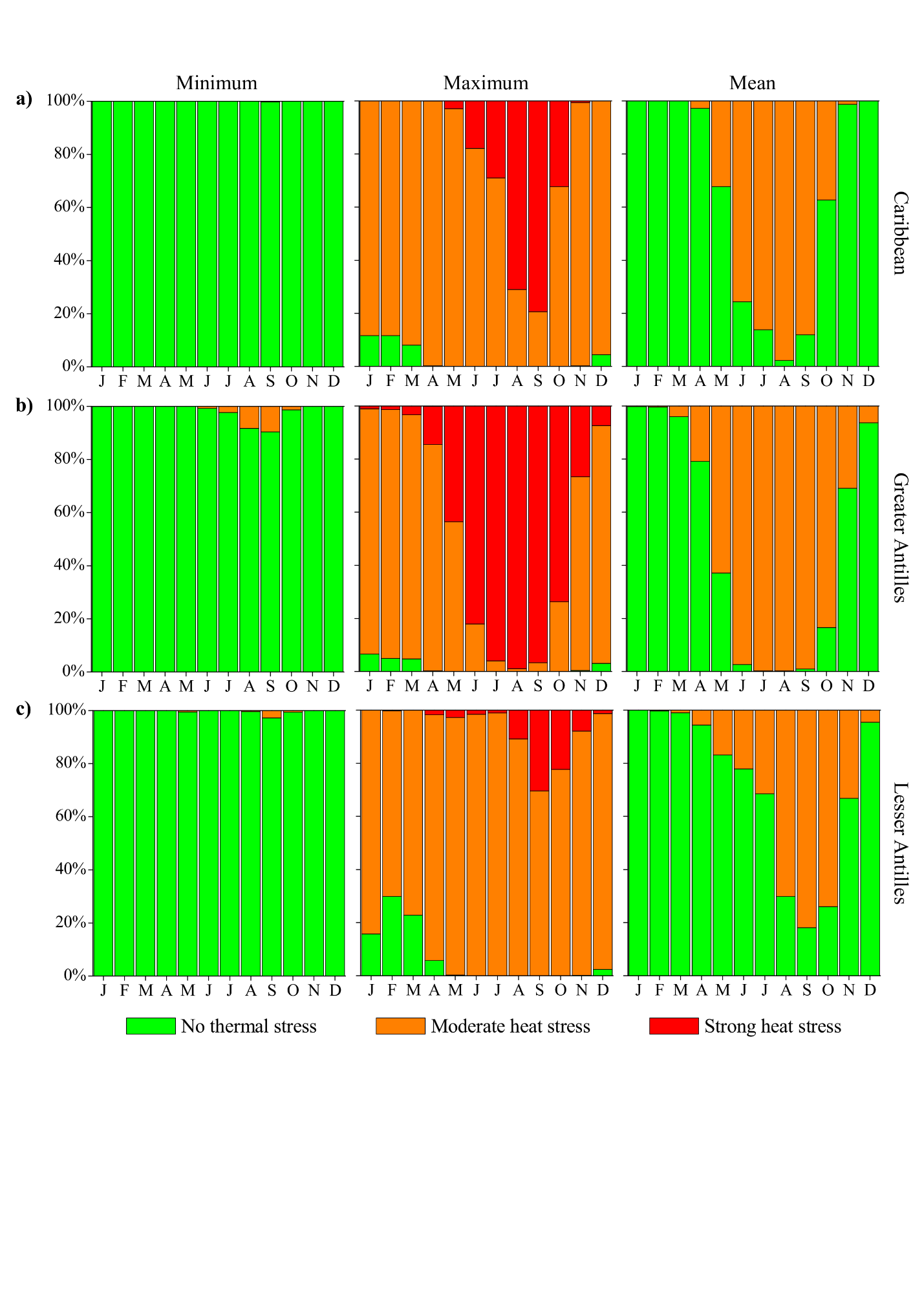


Figure S1: Monthly frequency of UTCI (Universal Thermal Climate Index) categories for the Caribbean region (a), the Greater Antilles (b) and the Lesser Antilles (c). The frequency refers to the percentage of days when daily minima, maxima and means fall in indicated UTCI categories over the 1980-2019 historical period.


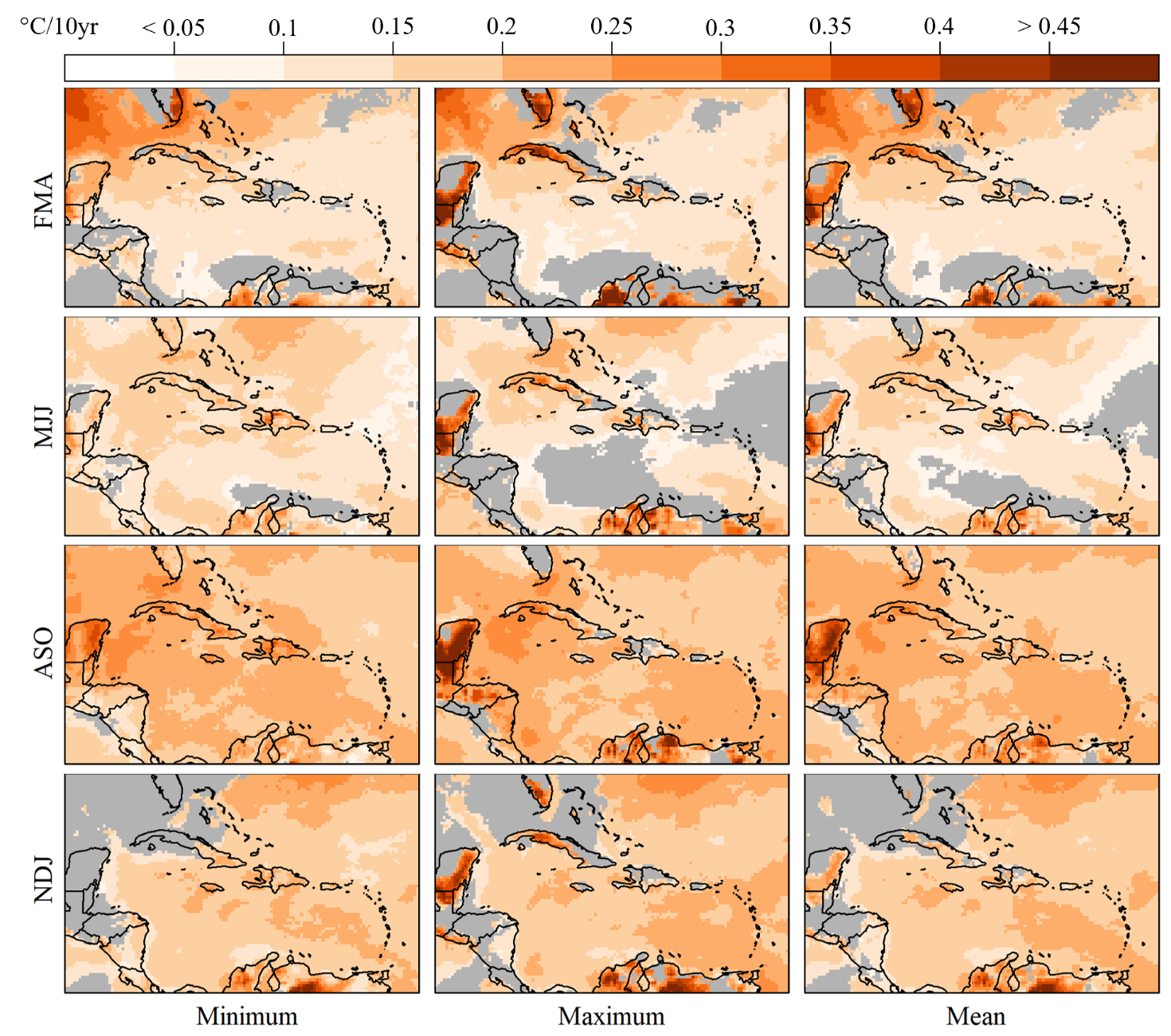


Figure S2: Spatio-temporal trends of 2m air temperature (minimum, maximum and mean) in the Caribbean region for the 1980-2019 period. Trends are represented by Sen’s slope coefficients and shown by seasons. Grey areas indicate grid cells where trends are not statistically significant according to the Mann-Kendall test (p ≥ 0.05).


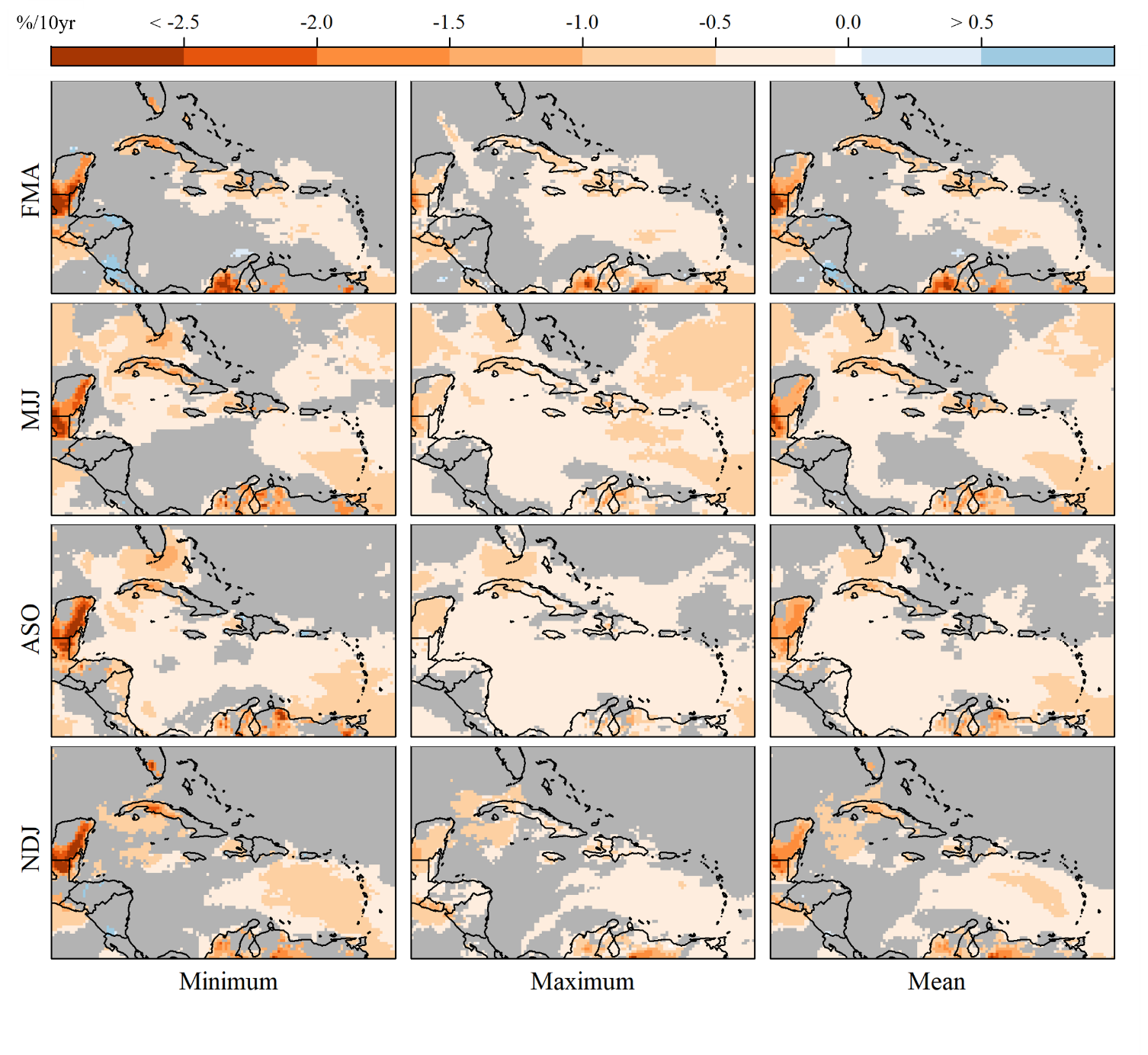


Figure S3: Spatio-temporal trends of relative humidity (minimum, maximum and mean) in the Caribbean region for the 1980-2019 period. Trends are represented by Sen’s slope coefficients and shown by seasons. Grey areas indicate grid cells where trends are not statistically significant according to the Mann-Kendall test (p ≥ 0.05).


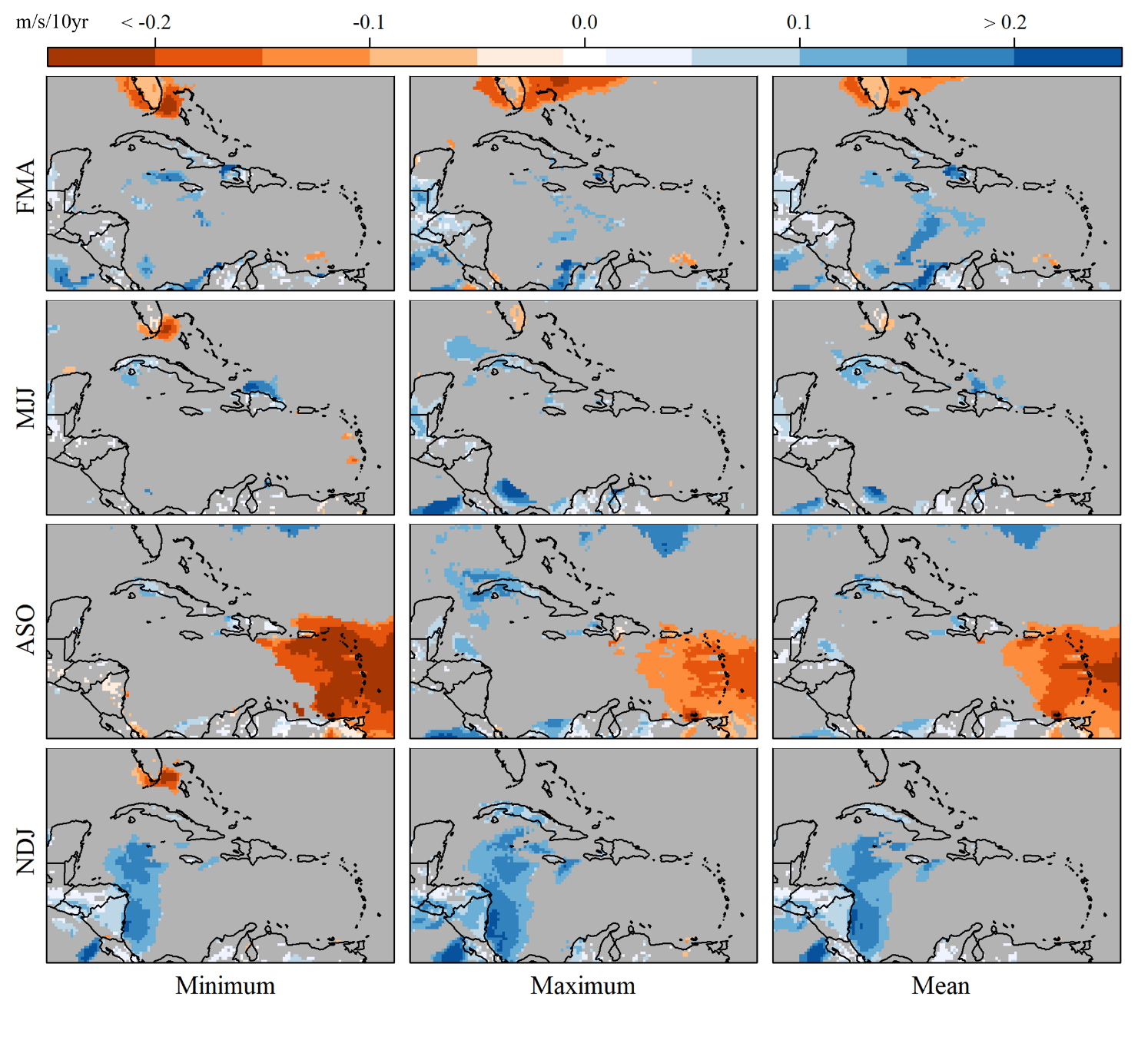


Figure S4: Spatio-temporal trends of 10m wind speed (minimum, maximum and mean) in the Caribbean region for the 1980-2019 period. Trends are represented by Sen’s slope coefficients and shown by seasons. Grey areas indicate grid cells where trends are not statistically significant according to the Mann-Kendall test (p ≥ 0.05).


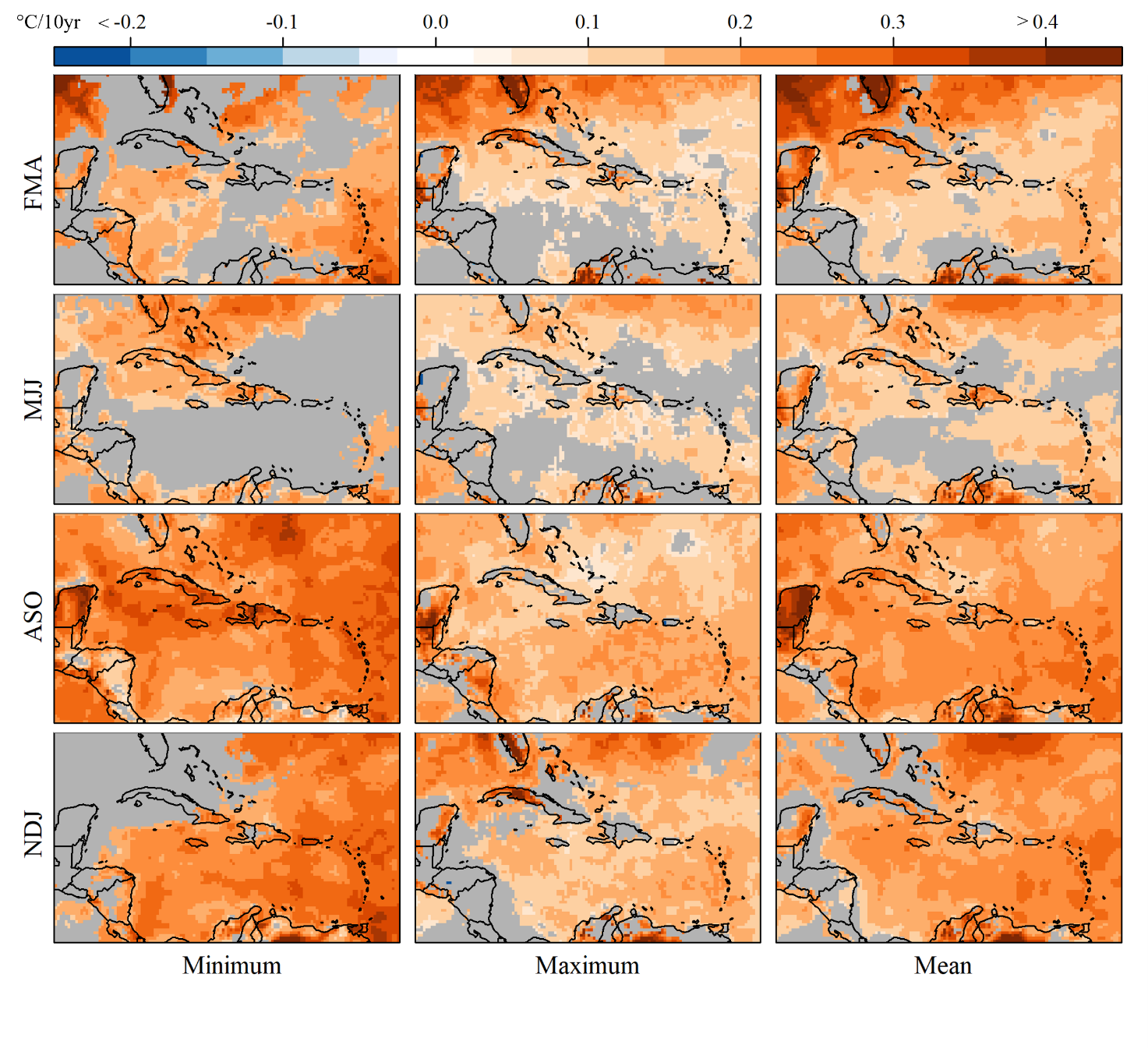


Figure S5: Spatio-temporal trends of mean radiant temperature (minimum, maximum and mean) in the Caribbean region for the 1980-2019 period. Trends are represented by Sen’s slope coefficients and shown by seasons. Grey areas indicate grid cells where trends are not statistically significant according to the Mann-Kendall test (p ≥ 0.05).


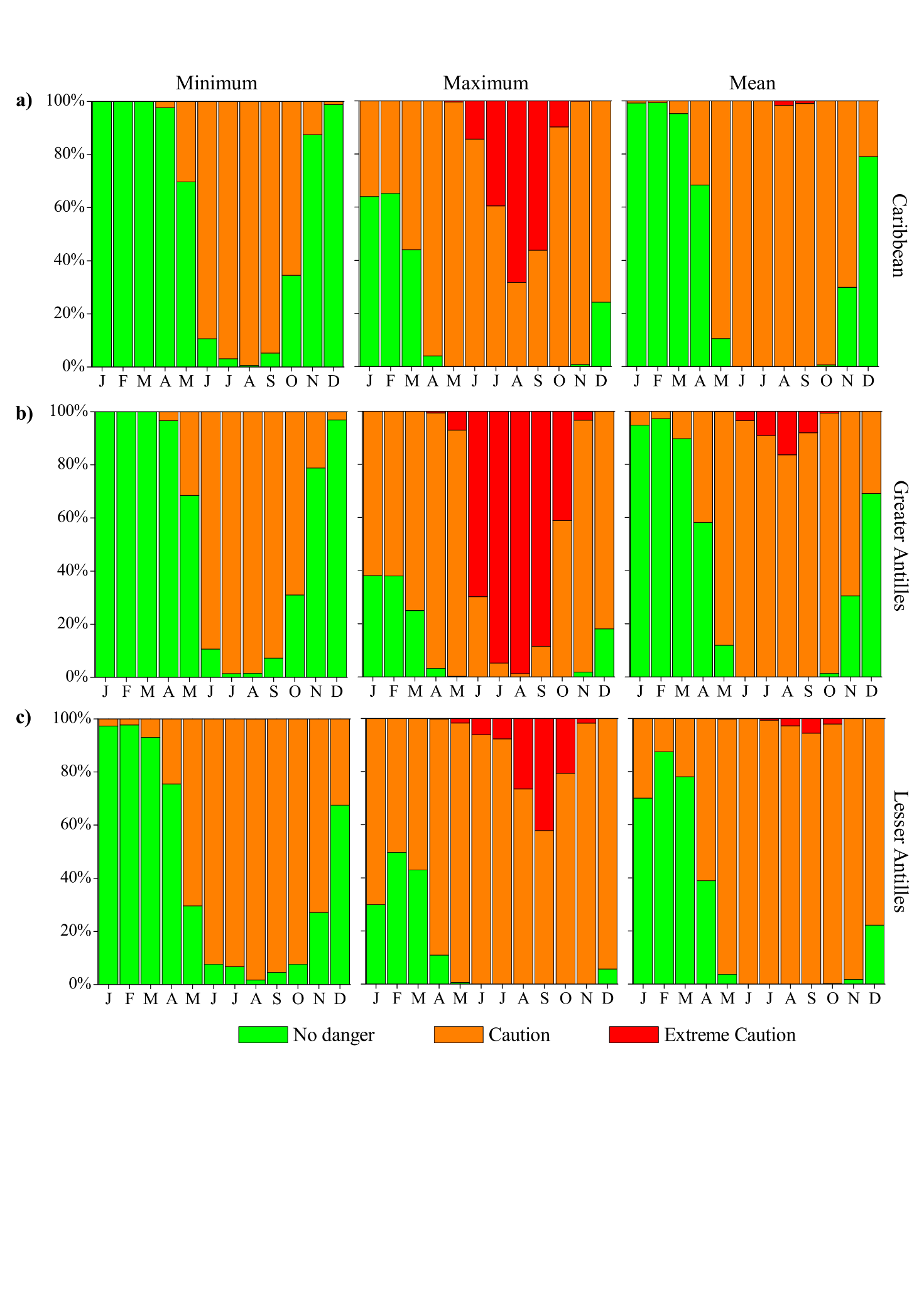


Figure S6: Monthly frequency of HI (heat index) categories for the Caribbean region (a), the Greater Antilles (b) and the Lesser Antilles (c). The frequency refers to the percentage of days when daily minima, maxima and means fall in indicated HI categories over the 1980-2019 historical period.


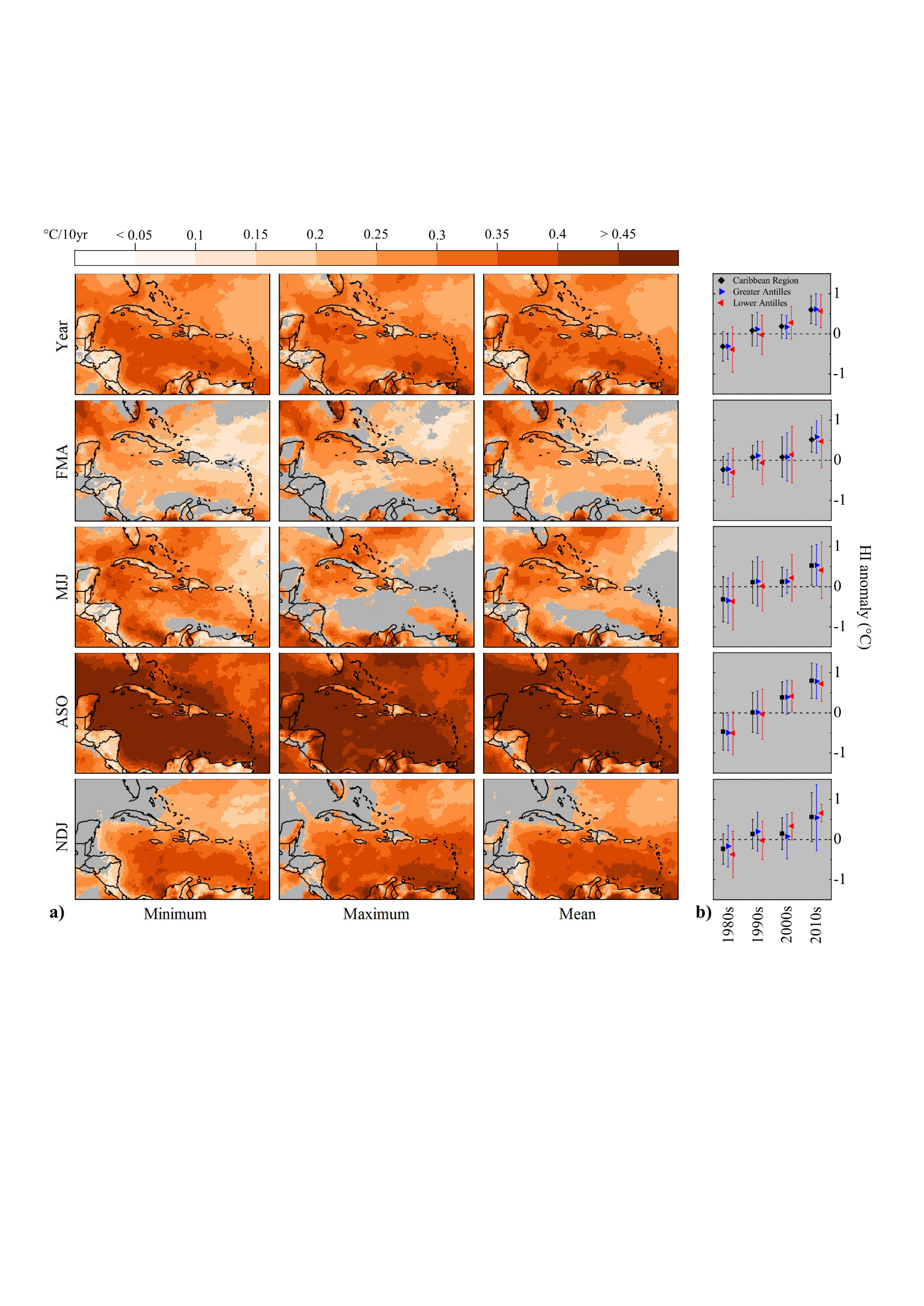


Figure S7: Spatio-temporal trends of the heat index (HI) in the Caribbean region. (a) Decadal trends, as represented by Sen’s slope coefficients, in minimum, maximum and mean HI for whole years and by seasons over the 1980-2019 period. Grey areas indicate grid cells where trends are not statistically significant according to the Mann-Kendall test (p ≥ 0.05). (b) Decadal anomalies of mean HI to the 1981-2010 climatological baseline by regions. Error bars represent one standard deviation.
